# Exploring Brand Hate and the Association Between Similar Competitor Offer and Brand Equity: A Moderated-Mediation Model

**DOI:** 10.3389/fpsyg.2020.533216

**Published:** 2021-01-15

**Authors:** Mudassir Husnain, Zanxin Wang, Petra Poulova, Fauzia Syed, Ahsan Akbar, Muhammad Waheed Akhtar, Minhas Akbar, Muhammad Usman

**Affiliations:** ^1^Department of Economics and Business Administration, University of Education Lahore, Faisalabad, Pakistan; ^2^School of Business and Tourism Management, Yunnan University, Kunming, China; ^3^Department of Informatics and Quantitative Methods, University of Hradec Kralove, Hradec Králové, Czechia; ^4^Faculty of Management Science, International Islamic University, Islamabad, Pakistan; ^5^International Business School, Guangzhou College of South China University of Technology, Guangzhou, China; ^6^Department of Management Sciences, COMSATS University Islamabad, Sahiwal, Pakistan

**Keywords:** brand equity, similar competitor offer, narcissistic personality, duplex theory of hate, brand hate

## Abstract

Using the assumptions of Sternberg (2003) Duplex Theory of Hate, the present study reveals the combined effects of similar competitor offer and narcissistic personality on brand equity through the underlying mechanism of brand hate. Specifically, we hypothesize that brand hate mediates the relationship between similar competitor offer and brand equity. Moreover, we propose that similar competitor offer and brand hate relationship are stronger for narcissistic individuals. By employing a multi-wave time-lagged research design, we collected data from a sample of (*N* = 338) dairy product consumers in Pakistan. The findings of moderated-mediation regression analyses indicate that (a) Brand hate mediates the relationship between similar competitor offer and brand equity; and (b) Narcissistic personality moderates a similar competitor offer and brand hate relationship such that a high similar competitor offer led to greater brand hate when narcissism was high. Furthermore, conditional indirect effects reveal that brand hate mediates the relationship between similar competitor offer and brand equity only with individuals exhibiting narcissistic personality traits. The current study offers great insights to managers that by managing similar competitor offer, they can manage the development of brand hate, which can subsequently effect brand equity. Moreover, by profiling customers on the basis of their personalities, marketing managers can effectively invest only in customers with positive tendencies. The current study is unique in that it highlights new avenues in existing research by extending the nascent domain of brand hate in consumer–brand relationships.

## Introduction

Consumers are the cornerstone of any enterprise’s success because it is they who decide whether or not to purchase the offered products or services and thereby determine the prosperity and existence of an organization (Kurajdová et al., 2015). It is particularly important for businesses to understand that negative information can affect their reputations and brand equity (Hegner et al., 2014). Previous studies have highlighted negative emotions’ association with failure to purchase certain food products (Johansen et al., 2011; Gutjar et al., 2015; Sosa et al., 2015). They concluded that negative emotions (aggression, disgust, annoyance, and disappointment) have significant impact on food product choices.

Dairy products constitute a large proportion of daily consumption in Pakistan (Geeta, 2015). Yet, growth of ultra-heat treated (UHT) milk, which has a shelf life of 6–9 months, is slow due to reports of poor quality adulteration (Zaheer, 2017), which caused consumer suspicion. In the food industry, hazards associated with dairy products, especially milk, have become serious concerns (Roncada et al., 2012). According to Ojha et al. (2017), milk adulteration is a dangerous and hazardous practice in developing countries including India, Pakistan, and Bangladesh. Due to reports of such adulteration, and experiences with poor quality, consumer behavior has turned negative toward UHT milk. The complicated emotions of dissatisfied consumers are more difficult to describe than the emotions of satisfied customers; such emotions result in consumer patronage reduction or cessation, complaining, or even boycotting of a product or service (Zarantonello et al., 2016).

It is obvious that negative consumption experiences resulting in consumer dissatisfaction usually give rise to negative emotions such as disgust, anger, fear, and contempt (Grégoire et al., 2009; Dimitriadis and Papista, 2010; Khan and Lee, 2014; Zhang, 2017). The extremely negative emotion hate, when directed toward a brand, produces deleterious effects on brand equity (Bryson et al., 2013; Zhang et al., 2019). According to Cãtãlin and Andreea (2014) faulty or potentially hazardous products may hamper or even irremediably damage brand equity.

The current study intends to shed light on some additional aspects such as similar competitor offer, explaining why consumers develop negative feelings about brands and the consequently adverse effects of these feelings on brand equity. Recent qualitative studies have narrowly focused on selective emotions that lead to crucial information but with piecemeal interpretations (Kavaliauskë and Simanavičiūtė, 2015); thus, a thorough understanding grounded in empirical evidence is much needed (Hegner et al., 2017). The current study adds to the literature on brand hate by answering strong theoretical calls for further investigation (Fournier and Alvarez, 2013; Park et al., 2013; Fetscherin et al., 2014), from a practical perspective, by examining how brand hate shows the negative impact of brand hate on consumption experience (Kucuk, 2019a).

To date, studies related to consumer negativity toward brands have mainly emphasized anti-consumption (Cherrier, 2009; Cromie and Ewing, 2009; Iyer and Muncy, 2009), anti-loyalty (Rindell, 2013), or even boycott (Yuksel and Mryteza, 2009) and have recently moved to brand avoidance (Liao et al., 2015; Hegner et al., 2017), brand rejection (Sandıkcı and Ekici, 2009; Nenycz-Thiel and Romaniuk, 2011), brand opposition (Wolter et al., 2016), anti-branding (Romani et al., 2015; Dessart et al., 2016), brand dislike (Romani et al., 2009), and brand hate (Bryson et al., 2013; Zarantonello et al., 2016; Hegner et al., 2017). Thus, to extend insights from this body of studies, the current study aims to unveil an important cause that instigates feelings of hate in consumers, which in turn harms brand equity.

Using clinical tests Bushman and Baumeister (1998) reveal that when individuals with narcissistic and/or egoistic traits are insulted or feel undervalued, they express deeper aggression than individuals without such traits. Individuals with narcissistic traits tend to demonstrate more aggression and hate when they think they are right even when their egos are not under threat from an adversary (Kucuk, 2019a). When such egoistic individuals see competitors of the brand offering the same products or feature, but with a higher level of prestige and prime importance, they are likely to begin developing negative feelings about their brand. Such narcissistic individuals are likely to develop hatred toward their own brand when they are unable to get something unique or extra that is not available from the competitors. The present study uses the narcissistic personality as a moderator between similar competitor offer and brand hate.

### Theoretical Background and Hypotheses Development

According to Sternberg (2003), hateful emotions (i.e., repulsion, disgust, anger, fear, and contempt) often results in violation of moral codes. He argues that combinations of these emotions may surface due to perceived violations of individual or social rights, and that for this reason may be seen as impending threats to their freedom, comfort, and self-preservation. Sternberg (2003) identified some typical hate stories designed around the topic of morality, in various ways. For instance, in Sternberg’s “criminal story,” the hated person or group is a criminal who has stolen something valuable from someone; in his “seducer-rapist story,” the rapist is an object of hate because he has hurt someone. Fitness and Fletcher (1993) point to these moral violations as a major reason for hate, stating that “hate was most often elicited by the perception that the subject had been badly treated, unsupported, or humiliated by the partner” (p. 945). They found that in hateful states, in addition to physically hurting, yelling, and throwing the target object, some people act coldly, or do nothing and ignore the situation.

### Similarity to Competitor Offer and Brand Equity

Brand and brand equity is the derivative effect the brand name has on consumer reaction toward the product or its marketing (Kotler, 2011; Gilal et al., 2018). In the last few decades, brand equity is an important intangible firm asset and has become a main attentive area of focus for practitioners and researchers (Farjam and Hongyi, 2015). Marketers are continuously creating and adopting strategies for building strong customer-based brand equity (Blackett, 1991; Emari et al., 2012). Growing interest in brand equity seems evident among the researchers, too, and has been widely discussed in literature with varying themes (Yousaf et al., 2017). Brand commitment reflects self-brand connections, and levels of congruence with consumers—the way they think and feel about themselves and others while using the branded item (Shuv-Ami, 2012). Stronger brands with positive brand equity ultimately have many benefits including high margins, powerful capability for communication effectiveness, brand extension opportunities, high consumer preference, and purchase intention (Buil et al., 2013; Su, 2016).

According to Brewer (1991), consumers have an inherent desire to balance their sense of individuality with their sense of inclusiveness. In certain conditions, similarity to competitor offer also generates a feeling of unwantedness thus generating a negative brand relationship. Sternberg (2003) found that negative emotions lead toward hate, which in turn has adverse effects on brand equity (Abid and Khattak, 2017). If a brand fails to meet customer requirements, it creates a negative impact that further adversely affects brand equity. Any violation of customer expectation can lead to negative emotions, which can lead to intentional avoidance of the brand; these violations may also include similarity to competitor offer. Romeo (1991) concluded that similar brand offers may cause decreases in brand equity. Product similarity to competitor offer has the power to enhance brand prestige if similarity with competitor’s product is low (Saleem et al., 2014). Based on the tenets of the duplex theory of hate (Sternberg, 2003), negative feelings sometime arise due to the targets diluted or unappealing personality. The duplex theory suggests, in particular, that perceptions of competitor similarity directly affect brand image and loyalty (Patterson and Smith, 2003; Keller, 2016). Based on these arguments the following is hypothesized:

***Hypothesis 1.*** *Similarity with the competitor is negatively related to brand equity.*

### Similarity to Competitor Offer and Brand Hate

Hegner et al. (2017) defined brand hate by building on the contentions of Sternberg (2003) Duplex Theory of Hate as, “a strong emotional responder of anger, contempt or disgust for a brand.” Past studies investigating the role of similarity to competitor offer suggest that a combination of other factors such as confusion and lack of knowledge also play in Wry and Castor (2017). Gierl and Huettl (2011) also postulate the existence of conditions under which high similarity is disadvantageous. Customers nowadays tend to give less patronage to brands that perform similarly to a competitor; this similarity can trigger feelings of anger and shame in customers (Romani et al., 2015; Sarkar and Sarkar, 2017) as they perceive themselves as the objects of cheating and betrayal (Sternberg, 2003). Negative emotions arise when any of the brand’s functions is interfered with or threatens the self-image of the consumer (Epstein, 1973), i.e., brand offers similarity to its competitor. In persistent consumer psychology literature, the betrayal–anger relationship is resilient where studies considered that betrayal is deemed much personal for which apology is not sufficient to repair the relationship (Jin et al., 2017; Da Motta and Hamilton, 2019; Lonergan et al., 2020). Not necessarily all transgressions result in brand hate; but elusively, when customer feels cheated in conjunction with unmet promises or indistinctive, i.e., similar offerings, such intense hateful emotion appears (Jain and Sharma, 2019). Through “love becomes hate effect,” it is probable that getting cheated by, or betrayed by, a brand has much adverse effects on consumer emotions, than mere customer disappointment (Tan et al., 2019, 2021). The duplex theory of hate (Sternberg, 2003) suggests that the inability to distinguish among multiple offerings is a frequent cause of consumers’ feeling cheated and betrayed (Romani et al., 2015). Thus, we hypothesize that:

***Hypothesis 2.*** *Similarity to competitor is positively related to brand hate.*

### Brand Hate and Brand Equity

Brands are considered as one of the most valuable assets for companies, and marketers adopt various strategies for building strong consumer-based brand equity (Blackett, 1991; Emari et al., 2012). A stronger brand with positive brand equity can ultimately contribute to high margins, communication effectiveness, brand extension opportunities, and high consumer preference and purchase intention (Shuv-Ami, 2012; Buil et al., 2013; Su, 2016).

Negative consumer emotions toward a brand, such as disregard, dislike, loath, and hate (Sampedro, 2017), and disliking and hate (Delzen, 2014), can vary in intensity. Kucuk (2019a) identifies three levels of brand hate: cold brand hate, cool brand hate, and hot brand hate and stated that these are the most intense level of negative feelings that a consumer experiences about a brand (Romani et al., 2012; Bryson et al., 2013; Abid and Khattak, 2017; Sarkar and Sarkar, 2017; Sampedro, 2017). Sternberg (2003) discussed hate in terms of three dimensions, repulsion and disgust, anger and fear, and devaluation through contempt.

In contrast to the concept of love, the concept of hate has not received sufficient attention in the literature (Abid and Khattak, 2017). Sternberg (2003) argues that there are many causes of hate and that hate is usually followed by several violations. His theory included dimensions including passion, anger, and negation of intimacy. It has been argued that negative emotions like disgust, anger, aversion, irritation, and disappointment generate brand hate (Sternberg, 2003; Preijers, 2016). Zhang (2017) has further confirmed that after a brand hate incident, brand equity is impaired. Public relations plans and other recovery strategies are essential to recovering lost brand equity.

In terms of behavioral outcomes, consumer hate is directed toward disputatious actions like brand avoidance or hateful feelings from mild (negative word of mouth or talking badly) to severe retaliatory behavior (Grégoire et al., 2009; Marticotte et al., 2016). Some suggest it is a consumer’s desire to punish brands for damage caused to them, or they try to distract themselves from the brand (Sampedro, 2017). Grégoire et al. (2009) describe hate as a form of desire for revenge, or desire for avoidance. Dissatisfied consumers may complain to the company or exit the relationship with the brand (Evanschitzky et al., 2011). The duplex theory suggests that emotional states like love, hate, and forgiveness provide reasons that form and develop attitude toward a brand (Sternberg, 2003; Han et al., 2015). Hence, we hypothesize that:

***Hypothesis 3.*** *Brand hates will be negatively related to brand equity.*

### Mediating Role of Brand Hate

According to Sternberg (2003) theory of hate, different components can cause brand hate and incite individuals to approach hated brands for revenge for the perceived violations committed by the brand. Violations of certain moral codes can trigger feelings of hate among individuals and propagate negative outcomes. Thus, the mediating role of brand hate in the relationship can be explained through the violation of moral codes; once experienced by consumers, such violations create hateful feelings compelling consumers to adopt anti-brand behaviors that damage brand equity. In case of similarity to competitor offer, the mediating role of brand hate also persists; contemporary consumers neglect those brands with similar offerings, those which trigger feelings of disappointment and anger (Romani et al., 2015; Sarkar and Sarkar, 2017) as they perceive themselves as having been cheated and betrayed by their brand (Sternberg, 2003). This can even result in a plea for brand avoidance resulting in reduced brand equity (Zhang, 2017). This customer behavior indicates a negative customer–brand relationship, which decreases the perceived value of the brand, and customers distract themselves from that brand.

Researchers agree that brand hate generated due to anger, disgust, and disappointment negatively affects consumer buying patterns, which leads to different behavioral responses like distraction (Sampedro, 2017). Previous studies have confirmed that brand hate mediates the relationship between brand transgressions and anti-brand activism (Romani et al., 2015), between brand avoidance and brand equity (Abid and Khattak, 2017), and between perceived betrayal and anti-brand action (Lee et al., 2013). Based on the above literature, we hypothesize that:

***Hypothesis 4.*** *Brand hate mediates the relationship between similarity to competitor offer and brand equity.*

### Moderating Role of Narcissistic Personality

According to Campbell et al. (2004) narcissistic personalities experience feelings of “deservingness” and are, in most situations, enthusiastic in their need to be dealt with fairly. In socio-psychology literature, hate is associated with the threatened egotism concept (Baumeister and Butz, 2005). These studies focus on threatened egoism in terms of contradiction between an individual’s positive and negative selves. Individuals can have these contradictory feelings easily, even when someone has threatened their positive self. Subsequently, these egoistic individuals show anger when their ego comes under attack or is criticized (Bushman and Baumeister, 1998). Internet and social media electronically bolster consumers’ expectations more than ever (Faulds et al., 2018), sometimes resulting in greater consumer disappointment, particularly in cases when the brand is indistinctive, i.e., a similar competitor offering (Ma, 2017). Thus, individuals become angry when seeing similar competitor offerings, which triggers extreme negative emotions toward these brands. Such hate can be magnified when consumers also have the egoistic tendencies and feelings of entitlement comprising the narcissistic personality (Kucuk, 2019a). Individual traits with varying altitudes have higher tendency to express hate/love toward some object so that the individual’s hateful emotions might be instigated due to their demanding nature and narcissistic personality (Kucuk, 2019b). Studies revealed that these less agreeable narcissistic individuals are more likely to fall in superiority complex, grandiosity, and wish to be admired (Thomaes et al., 2013; Turel and Gil-Or, 2019). Such narcissist consumers perceive self-brand incongruence or loss of self-brand connection as an outcome of diluted, i.e., brand indistinctiveness. Also, brand meaning is an apparent topic in anti-consumption literature than ever (Fetscherin, 2019; Kucuk, 2019b). It is therefore probable that an individual with narcissistic tendencies express deep hatred for a brand that might be perceived similar to that of a competitor and take it as an act of betrayal on the part of the brand. Based on the Sternberg (2003) theory of hate, when highly narcissistic consumers observe that their brand offers a product similar to a competitor’s, the intensity of their brand hate increases. Thus, it is hypothesized:

***Hypothesis 5.*** *Narcissistic personality moderates the relationship between similar competitor offer and brand hate, such that the positive relationship between similar competitor offer and brand hate will be stronger when consumers are high in narcissistic personality and vice versa.*

### Conditional Indirect Relationships

As argued in the previous section, narcissistic personality moderates the relationship between similar competitor offers and brand hate, and it is likely that narcissistic personality will conditionally impact the strength of the indirect relationship between similar competitor offer and brand equity, thus eliciting moderated mediation. As it was hypothesized that the relationship will be stronger or vice versa between similar competitor offer and brand hate based on varying values of moderation, additionally, the indirect relationship will also conditionally impact the varying values, i.e., high and low of moderators. When individuals have highly narcissistic personalities, they are more likely to lean strongly toward the brand equity through brand hate.

***Hypothesis 6.*** *Narcissistic personality moderates the positive and indirect effects of similar competitor offer on brand equity (through brand hate). Specifically, brand hate mediates the indirect effects when narcissistic personality is high or vice versa.*

## Research Methodology

**FIGURE 1:**
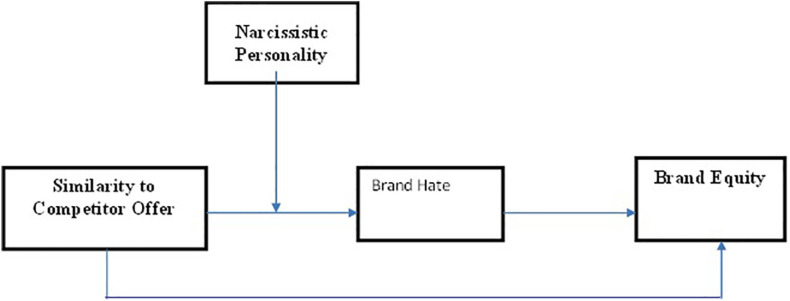
Conceptual framework.

The study intends to examine the mediating role of brand hate in the relationship between similar competitor offer and brand equity. Furthermore, it investigates the moderating role of narcissistic personality in the relationship between similar competitor offer and brand hate (see Figure 1). English is the official language of higher education and business in Pakistan; consistent with prior studies (Abbas et al., 2014) conducted in Pakistan, the present research administered surveys in English. Data were collected from individuals who are UHT milk users. The tetra pack products were chosen because similar product offerings are ever growing in this industry. This is problematic due to the fact that consumers are continuously bombarded with the advancement and sophistication of high technology (Russell and Kellershohn, 2018). Such similar tetra brand penetration has been often observed in various reports released by PCSIR (2019). The target population for the current study comprises tetra pack consumers. The respondents approached were individuals working in educational institutes of northern Punjab, Pakistan, using non-probability convenience sampling technique. Convenience sampling technique was deemed suitable for the present study, due to unavailability of a large pool of consumers over multiple time lags, costs associated in accessing the respondents, and time-consuming nature of research design.

The survey was administered in three rounds/time-lags, 3 weeks apart. Majority of the respondents chosen for the study were from the education sector, and surveys were limited to those who have Internet access (Bennetts et al., 2019). For instance, in time 1, offline surveys were distributed among participants, and to obtain a quicker response with convenience to both researchers and respondents, online surveys were circulated in time 2 and time 3 to the same respondents. Studies such as longitudinal/time waved in social sciences are considered more convenient or tradition (Cole and Maxwell, 2003). The independent variables and the moderating variable (similarity to competitor offer and narcissistic personality) were tapped in time 1, the mediating variable (brand hate) was tapped in time 2, and the dependent variable (brand equity) was tapped in time 3.

Each survey was accompanied by a cover letter explaining the research objective and highlighting that the results would help researchers understand the mechanisms of hate. Participants were ensured complete confidentiality and told that there was no right or wrong answers, to diminish the risk of acquiescence or social desirability biases (Spector, 2006). At time 1, respondents were asked to think about top five leading UHT brands and mention their names based on their perceptual evaluation. After that, they were required to respond the appropriate option from the questionnaire items. For the present study’s time-lagged design, modest compensation was given to respondents to ensure their participation. All contacted participants’ records were maintained in an excel sheet at each time wave. Moreover, individuals were informed that only aggregate data findings would be reported. Researchers proposed that a sample ranges from 3 to 500 and is considered sufficient in consumer behavior research (Kaden, 2006), and relatively enough to conduct time lagged/temporal studies (Boomsma, 1983). To maintain generalizability and precision in our findings, at time 1, a total of 600 questionnaires were distributed out of which 450 properly filled out questionnaires were received, yielding a response rate of 75%. At time 2, of the 450 respondents from time 1 who were re-contacted, 381 returned completed questionnaires. Finally, in time 3, a total of 338 useable surveys were received resulting in a response rate of 56%. Among them, 71% were 21–29 years of age, their average qualification was master, and most of them were students (76%).

### Measurements

As presented in Table 1, the constructs used in our study were drawn and adapted from many sources, i.e., Su (2016), Hegner et al. (2017), Stokburger-Sauer et al. (2012), and Kansi (2003). The scale for “Brand equity” was collected from the study of Su (2016). Items measuring “Brand hate” was adapted and modified from Hegner et al. (2017). The scale for “similar competitor offer” was created by Stokburger-Sauer et al. (2012) with modification as per the study’s requirements. A short version of the “Narcissistic personality” measurement scale was taken from Kansi (2003). All the selected items were measured on a five-point Likert scale (strongly disagree = 1, disagree = 2, neutral = 3, agree = 4, strongly agree = 5).

**TABLE 1:** Instrument details.

Constructs	Author	Number of items
Brand equity	Su, 2016	13
Brand hate	Hegner et al., 2017	6
Similar competitor offer	Stokburger-Sauer et al., 2012	4
Narcissistic personality	Kansi, 2003	6

## Results

The demographic profile of the respondents shows that 178 male and 160 females (52.6 and 47.3%, respectively) participated in the study. Most of the respondents have qualified for graduation and master’s degrees (32.2 and 38.7%, respectively). The majority of the sample falls between the ages of 21 and 29, which is 71.3% of the total sample. Most of the respondents (47.0%) have been using dairy product for more than 3 years (see Table 2).

**TABLE 2:** Demographics.

Demographic	Classification	Frequency	Percentage
Gender	Male	178	52.6
	Female	160	47.3
Education	Matric/O-levels	12	3.5
	Intermediate/A-levels	39	11.5
	Graduation	109	32.2
	Masters	131	38.7
	MS/Ph.D.	47	13.9
Profession	Student	171	50.5
	Professional	127	35.5
	Self-employed	29	8.5
	Other	11	3.2
Age	Under 20	11	3.2
	21–29	241	71.3
	30–39	73	21.5
	40–49	5	1.4
	50+	8	2.3
How long have you been a dairy products user?	1 year	62	18.3
	2 years	73	21.5
	3 years	44	13.0
	More than 3 years	159	47.0

### Common Method Analysis

For addressing the common method bias, we used several diagnostic analyses. First, we used a time lag approach by which data related to similar competitor offer and narcissistic personality trait were conducted in T1; after a 3-week interval, we conducted brand hate data in T2, and in T3, we collected brand equity data after a 3-week interval (Podsakoff et al., 2003). Second, common method bias is less salient in models that investigate the moderating effect because it is difficult to guess by the respondent, and that will decrease the likelihood of spurious findings (Brockner et al., 1997; Simons and Peterson, 2000). Third, to ensure that common method bias was not a problem for this study, the study assessed its possible occurrence with Herman’s one-factor test. Harman’s one-factor test in CFA produce a poor model fit for the sample: χ^2^ (df) = 3,039.52 (582); *p* < 0.001; RMSEA = 0.18; NNFI = 0.29; CFI = 0.34. This ensures no chance of bias factor being likely to explain variances in measures.

### The Measurement Model Evaluation

The measurement model was evaluated using convergent validity, reliability, discriminant validity, and fit indices. It has the benefit of using a method for dealing with the dependence of multiple and interrelated relationships and the statistical efficiency to examine individuals possessing subjective assessments for directly unobservable concepts in terms of number of observable components (Hair et al., 1998). Reliability was evaluated by Cronbach’s alpha test on all items. The values should be greater than 0.70 (Nunnally, 1978). The results showed that the values ranged from 0.701 to 0.813.

### Convergent Validity

In order to test convergent validity, the average variance extracted (AVE), composite reliability (CR), and factor loadings of all items were considered. Convergent validity indicates how different items measure a single construct. According to Hair et al. (1998), the minimum value for all factor loadings should be 0.5. The value of AVE should be greater than 0.5 (Fornell and Larcker, 1981). According to the results, the factor loadings for all items ranged between 0.71 and 0.90. Four items were excluded, two from brand equity and two from brand hate. The CR of all constructs was greater than 0.7. The AVE for each construct was greater than 0.5 (see Table 3).

**TABLE 3:** Convergent validity.

Constructs	Items	Factor loading	Composite reliability	Average variance extracted	Cronbach α
Brand awareness	BA2	0.85	0.892	0.734	0.778
	BA3	0.86			
	BA4	0.86			
Brand association	BAS1	0.79	0.817	0.599	0.798
	BAS2	0.71			
	BAS3	0.82			
Brand loyalty	BL1	0.81	0.823	0.608	0.813
	BL2	0.77			
	BL3	0.75			
Perceived quality	PQ1	0.72	0.811	0.561	0.789
	PQ2	0.76			
Brand hate	BH1	0.78	0.808	0.538	0.781
	BH2	0.84			
	BH3	0.86			
	BH4	0.77			
Similar to competitor offer	SCO1	0.90	0.710	0.585	0.701
	SCO2	0.75			
	SCO3	0.81			
Narcissistic personality	NP1	0.79	0.782	0.522	0.778
	NP2	0.71			
	NP3	0.74			
	NP4	0.85			
	NP5	0.73			
	NP6	0.81			

### Discriminant Validity

As presented in Table 4, discriminant validity is ensured if the square root of the AVE for each construct is greater than its correlation with each construct. The discriminant validity of all constructs was established.

**TABLE 4:** Discriminant Validity.

	Similar competitor offer	Narcissistic personality	Brand hate	Brand equity
Similar competitor offer	**(0.706)**			
Narcissistic personality	0.075	**(0.650)**		
Brand hate	0.071	0.130	**(0.733)**	
Brand equity	–0.097	–0.509	–0.133	**(0.650)**

*The values in brackets are square root of (AVE).*

The values in brackets are square root of AVE.

### Fitness Measures

**FIGURE 2:**
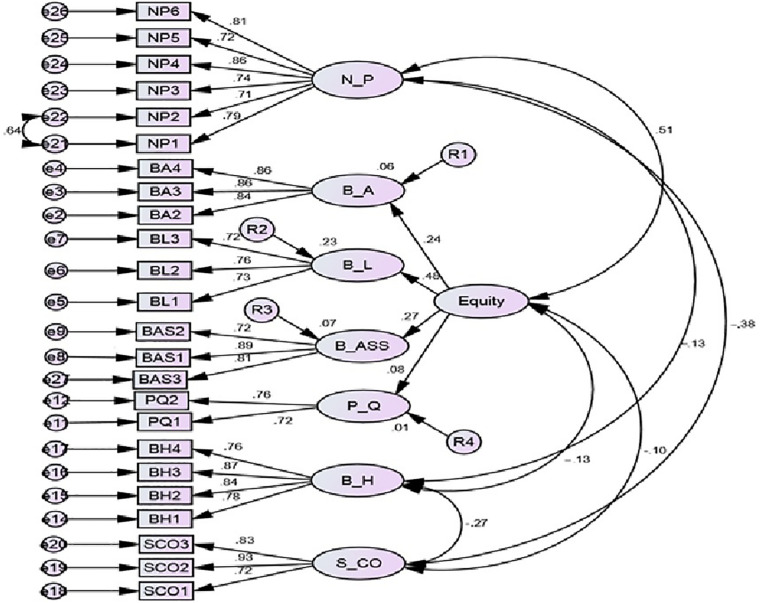
Structural model

Chi-square/df, goodness-of-fit index (GFI), comparative fit index (CFI), Tucker–Lewis index (TLI), and root mean square error of approximation (RMSEA) were used to check the fitness of the model and indicated a good fit (Chi-square/df = 2.016, GFI = 0.910, CFI = 0.939, TLI = 0.927, RMSEA = 0.053). According to Bentler and Bonett (1980), the fit measures should have a score above 0.9. The value of Chi-square/df should be less than 3. According to Hair et al. (1992), the RMSEA should be less than 0.08 (see Table 5 and Figure 2).

**TABLE 5:** Fit indices for measurement model.

Fit measures	Result
Chi-square/df	2.016
Goodness-of-fit index (GFI)	0.910
Comparative fit index (CFI)	0.939
Tucker–Lewis index (TLI)	0.927
Root mean square error of approximation (RMSEA)	0.053

### Structural Model Evaluation and Hypothesis Testing

**FIGURE 3:**
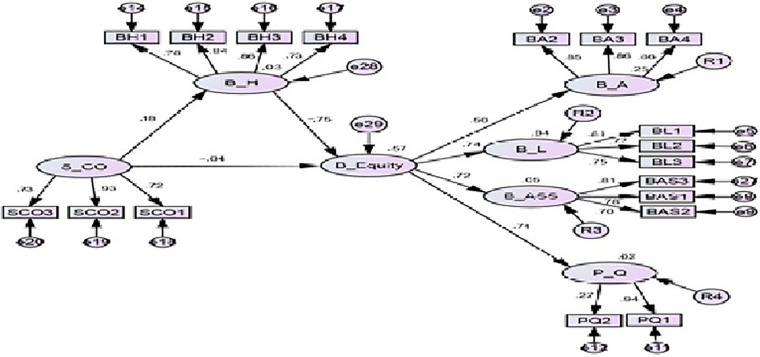
Path model.

The relationships among different variables were examined through the structural equation model. The results of fit indices (Chi-square/df = 2.542, GFI = 0.906, CFI = 0.911, TLI = 0.894, RMSEA = 0.057) indicate a good fit (see Table 6 and Figure 3). Results of hypotheses testing are shown in Table 7. Hypotheses were assessed based on critical *t*-value, with a value greater than 1.96, representing a significant path. The results suggest that similar competitor offer has a positive significant effect on brand hate (β = 0.18, *t* = 5.60, *p* < 0.001), and brand equity (β = −0.04, *t* = −5.73, *p* < 0.001). Therefore, Hypothesis 1 (H1) and Hypothesis 2 (H2) are supported. Also, brand hate has a positive significant relationship with brand equity (β = −0.75, *t* = −3.92, *p* = 0.002). Hence, Hypothesis 3 (H3) was substantiated.

**TABLE 6:** Fit indices for structural model.

Fit measures	Results
Chi-square/df	2.542
Goodness-of-fit index (GFI)	0.906
Comparative fit index (CFI)	0.911
Tucker–Lewis index (TLI)	0.894
Root mean square error of approximation (RMSEA)	0.057

**TABLE 7:** Structural relationships.

Hypotheses	Paths	Beta	Standard error	*T*-values	Result
H1	Brand equity ← similar competitor offer	–0.04	0.091	−5.734**	Supported
H2	Brand hate ← similar competitor offer	0.18	0.032	5.606**	Supported
H3	Brand equity ← brand hate	–0.75	0.062	−3.920*	Supported

Critical t-values: *1.96 (significance level=5%), and **2.58 (significance level=1%), N=338.

### Testing Mediation

In addition to the direct effect, the current study examines the mediating role of brand hate in the relationship between similar competitor offer and brand equity. As indicated in Table 7, similar competitor offer has a significant relationship with brand equity, fulfilling the first condition. Similarly, similar competitor offer has significant relationship with brand hate, which indicates that the second condition was met. Furthermore, the effect of brand hate on brand equity indicates a significant relationship, thereby meeting the third condition. The final step is the addition of the mediator brand hate to determine whether it reduces the effect size or not. With the addition of brand hate, the indirect effect was (β = −0.131; *p* < 0.05), indicating that there is a mediation effect, and that the direct effect between similar competitor offer and brand equity becomes insignificant (*p* = 0.396) thus confirming the mediating role of brand hate in the relationship. Hence, H4 is supported.

**FIGURE 4:**
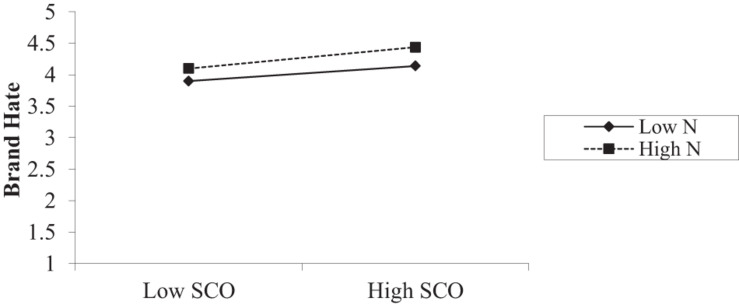
Interaction plot.

Hypothesis 5 (H5), predicted that narcissism will moderate the relationship between similarity to competitor and brand hate. The results in Table 9 demonstrate that the similarity to competitor and narcissism interaction was significant for brand hate (*B* = 0.19, SE = 0.04, *p* < 0.001). The bootstrap results proved that the conditional direct effects of IV on DV exist for varying levels of the moderator particularly in the case of high moderator (high narcissistic personality) for brand hate (see Table 8). A simple slope analysis (Aiken et al., 1991) interaction plot also supported that narcissistic personality moderates the relationship. The nature of the relationship between similarity to competitor and brand hate changes as the function of narcissism changes (low, moderate, and high). Figure 4 depicts that, in line with our H5, the positive relationship between similarity to competitor and brand hate was stronger (and positive) when narcissism was high (β = 0.16, *t* = 2.52, *p* < 0.001), whereas it was insignificant when narcissism was low (β = 0.006, *t* = 0.101, *ns*). Thus, H5 was supported.

**TABLE 8:** Moderated regression analysis results.

Predictors	Narcissistic personality			
	
	*R*	*R*^2^	Estimate	SE
Step 1	0.33***	0.11***		
Constant			6.19***	1.002
SCO			−0.69*	0.26
CV			−0.56**	0.25
Step 2	**Δ*R*^2^**	0.03*		
SCO×CV			0.19**	0.04

**Conditional direct effects of X on Y at values of moderator (i.e., narcissistic personality, N) (slope test results)**				

**N**	**Effect**	**Boot SE**	**LLCI**	**ULCI**

N low	0.006	0.06	–0.12	0.13
N moderate	0.08	0.06	–0.03	0.20
N high	0.16*	0.06	0.04	0.28

*n* = 338. Unstandardized regression coefficients are reported. Bootstrap sample size=5000; LL, lower limit; CI, confidence interval; UL, upper limit; SCO, similarity to competitor offer; N, narcissistic personality; BH, brand hate. *p < 0.05, **p < 0.01, ***p < 0.001.

**TABLE 9:** Regression results for conditional indirect effects (moderated mediation).

Independent variable	Mediator	Dependent variable	Moderator (NP)	Indirect effect	SE	95% Bootstrap CI; (LLCI, ULCI)
Similarity to competitor offers	Brand hate	Brand equity	High	−0.02***	0.02	(−0.052, −0.004)
			Medium	−0.01*	0.01	(−0.04, 0.001)
			Low	−0.00**	0.01	(−0.025, 0.016)

*N* = 338. Unstandardized regression coefficients are reported. Bootstrap sample size=5,000. SE, standard error; LL, lower limit; CI, confidence interval; UL, upper limit. *p < 0.05, **p < 0.01, ***p < 0.001.

Table 9 reported the results for conditional indirect effects (Hypothesis 6), whereby narcissistic personality moderates the indirect effects of similarity to competitor offer on brand equity through brand hate. In particular, we hypothesized that brand hate mediates the indirect effects when narcissism is high and not when it is low. Thus, we confirmed the conditional indirect effects of similarity to competitor offer on brand equity at three different values of narcissism (see Table 9), the mean (0.00), one standard deviation above the mean (0.40), and one standard deviation below the mean (−0.40).

Normal theory analyses show that the conditional indirect effects (based on moderator values at one standard deviation above the mean was negative and significantly different from zero).

Bootstrap confidence intervals confirmed these findings. The indirect and negative effects of similarity to competitor offer on brand equity through brand hate were present under levels of high narcissism (indirect effect = −0.02, *p* < 0.001) (bootstrap 95%). CI did not include zero (−0.05, −0.004), but not when narcissism was low (indirect effect = −0.009, *p* = *ns*) 95% CI containing zero (−0.02, 0.016). Thus, Hypothesis 6 was fully supported.

## Discussion

Brand hate is one of the key themes in the growing body of consumer–brand relationship literature and is gaining attention from academia and practitioners alike. The damaging effects of brand hate on organizations are likely to impose high costs on firms to ensure their survival. The literature discussing the negative side of the consumer–brand relationship mostly emphasized emotions such as anger, disgust, and disappointment (Shaver et al., 1987) and neglected other affective reactions such as brand hate (Zarantonello et al., 2016). Thus, the present study contributes to the nascent domain of brand hate, as it is apparent that the literature on antecedents and outcomes of brand hate is not sufficient and demands more clarity (Zhang, 2017).

Additionally, similarity to competitor offer is also gaining a place in marketing literature (Gray et al., 2017) due to the fact that it costs for firms, to consumer morality, and subjective notions of justice and entitlement (Wolter et al., 2016). Therefore, the present research adds to the growing evidence by revealing the effects of similar competitor offer on brand equity through brand hate. Similarity to competitor offer is considered as unmoral behavior and social misconduct (Trump and Newman, 2017). Klein and Dawar (2004) concluded that brand equity may protect against consumers’ switching because it is built by meeting customer’s needs and requirements. Negative customer–brand relationships such as brand hate can damage the company that owns the brand (Fournier and Alvarez, 2013).

The current study’s findings reveal that negative feelings due to similarity to competitor offer can be a consequence of several factors. For instance, a brand introduces a product similar to that of a competing brand, which leads to a loss of individuality for customers who perceive this as a negative value (Lee et al., 2009). Consumers tend to usually favor brands with which they perceive to have congruence with and match their personality (Sung and Huddleston, 2018). Therefore, current study findings are in line with past literature Ali et al. (2020) that narcissistic individuals tend to show deep anger when their ego is threatened, such as by seeing competitors with similar product offers. In such scenarios, customers perceive that their personal brand has no distinctiveness over the competitors. Thus, such individuals immediately stop patronizing the brands that resemble other (competitor’s) offers because they perceive this as misfit with one’s own self.

Thus, market research suggests to carefully review competitors’ brands in order to avoid the loss due to similarity with competitor’s offer. This study provides a comprehensive perspective on negative customer–brand relationship and brand equity.

Dissatisfied consumers are becoming the utmost source of insights for both marketers and academics. The current study identifies how the dynamics of brand hate evolve and effect changes in brand equity due to these negative emotions. There is a strong rivalry among the UHT milk firms, comprising a vast base of customers who usually seek benefits in terms of quality. Due to many competing UHT brand options, customer switching is likely probable if they did not get the desired benefits. Companies affirm in parallel ways that loyalty enhances brand equity and helps to gain sustainable competitive advantage, while negative emotions can spoil brand image.

The current study supports the contention that consumers develop feelings of hate toward their UHT milk brand if the brand did not meet their expectations whether in quality, customer self-image, or along moral and ethical lines. Dynamics of brand hate such as similarity to competitor’s offer are the basic negative factors that can accumulate until the customer intentionally avoids the brand. Furthermore, any brand of UHT milk that does not render the expected utility (e.g., similarity to competitor’s offer) creates a diluted image, which often leads to brand hate, thus harming brand equity.

The study’s findings also demonstrate that brand hate mediates the relationship between similarity to competitor’s offer and brand equity. In line with earlier research (Van der Kaap-Deeder et al., 2016), *distinctive* brand attributes promote positive outcomes, i.e., satisfaction, loyalty, and brand equity. Similarly, in the dairy sector, acceptance of a brand does not merely depend on taste, but on other factors as well, for instance, other intangible and tangible attributes of brands such as unique positioning, which can result in increased brand equity (Islam et al., 2019).

It is thus imperative to effectively reduce such hateful feelings, which can deteriorate brand equity. This can be done by reducing the gap between product performance perception and actual product performance (Lee et al., 2009). Thus, it is necessary to devise a mechanism to adequately deal with customer anger, which developed due to the negative customer–brand relationship generated by perceived attacks against their self-image through the malpractices of organizations.

Moreover, the present study supports the notion that not all consumers react equally to the firm’s immoral acts such as similarity to competitor’s offer (Kucuk, 2019a; Gray et al., 2017; Akhtar et al., 2020a). Based on the available literature and to the best of our knowledge, narcissistic personality has not been investigated between similarity to competitor’s offer, brand hate, and brand equity together. Consumers high on narcissism are more likely to fall in brand hate compared to consumers low on this trait.

We found sufficient support for our moderated mediation mechanism where similarity to competitor’s offer for people with narcissistic personality traits results in more brand hate. Particularly, brand hate mediated the negative effects of similar competitor offer on brand equity. This indirect effect was specifically more pronounced for consumers with narcissistic personality traits. The present research makes a unique contribution as it provides temporally separated longitudinal data from users of UHT milk. Employing three-wave data significantly reduced chances of common method bias and multicollinearity issues particularly in the complex moderated mediation framework. Furthermore, we employed the Preacher and Hayes (2004) process technique, which utilizes bootstrapped confidence intervals for approving moderated mediation, simple mediation, and moderation effects simultaneously.

### Implications for Managers and Marketers

From a marketing standpoint, firms should keenly assess causes of consumer’s brand attitude changes. In marketing literature, food consumption is normally associated with consumer wellbeing. Consumer perceptual evaluation of poor quality and hazardous UHT tetra pack brands results in abandonment or even hateful emotions for those brands. Therefore, there is a timely need to manage perceptions of tetra pack brands that aids customer and societal wellbeing. The current study recommends some strategies and tactics to handle brand haters. First, it is essential for companies to continuously monitor consumers’ relationship with front desk personnel, customer service centers, and brand social media pages and other web presences. To keep their fingers on the pulse, internal and external tracking systems are imperative for coping with consumer–brand relationships efficiently and effectively. Such strategies will likely prove beneficial to firms with dissatisfied customers. The current research offers significant contribution in both marketing practice and literature by providing a comprehensive understanding of brand hate.

Second, negative emotions triggered in different intensities need to be managed carefully. To deal with similarity to competitor’s offer, companies should deliberately evaluate the degree of similarity between competitors’ brand and firm’s own brand. Moreover, efforts must be made to effectively manage angry customers to lower the intensity of their negative emotions toward the firm’s brand.

Therefore, the findings that emerged from the empirical investigation of this study are fruitful for marketers as well as suppliers of products. Sometimes, an honest mistake can be perceived as a brand transgression; therefore, managers and marketers should keep monitoring public relations departments to assess whether problems are being perceived as intentional or unintentional then develop some strategies that mitigate the damage caused. In today’s marketplace, similarity to competitor’s offer is a major reason for the brand hate that consequently triggers negative behaviors. For managers and marketers, selecting a real and well-conceived target market is advisable; by doing so, firms can offer the right type of product to the right customers and ensure progressive brand equity.

### Limitations and Future Research Directions

Though the current research is not free from limitations, those limitations can be focused upon in future studies. That consumer negativity toward brand is gaining attention implies that there are interlinked areas that require examination by researchers; future studies should examine the role of personality traits responsible for propagating the feeling of hate (Sternberg, 2003; Hegner et al., 2017; Akhtar et al., 2020b). As argued by Sternberg (2003), in addition to retaliation and revenge, ignoring or doing nothing with regard to the hated object might be an individual’s response. In consumer research, it would be interesting to see if “*status quo*” is an additional factor in brand hate. Future studies can expand our framework and also recommend other mechanisms and situations under which similarity to competitor offer influences some other outcomes through brand hate. For instance, it is advisable to study other dispositional traits such as anger, negative affectivity and the Big 5 emotions, which might take similarity to competitor offer being threatening for one’s self-image. Future studies can also investigate other behavioral and cognitive outcomes of similarity to competitor offer and brand hate such as alienation, anger, retaliation, and investigate why and how similarity to competitor offer and brand hate shape them up.

The present study only targeted the UHT milk industry. The present study could also be replicated in other sectors like FMCGs, smart phones, and electronics. Along with this, future studies should target brand services to see if similarity to competitor offer creates the same intense reactions of brand hate and subsequent decline in brand equity similarly across the different service industries or not.

As the current research adopts a time lagged research design, it cannot be considered a pure longitudinal design because the study variables were measured in three separate time periods. Future researchers can employ pure longitudinal designs to further validate our findings here. The sample considered in this research comprised male and females in public and private universities, colleges, and schools, and was obtained through convenience sampling. Enhancements in sample selection and sampling technique can be performed to acquire more generalizable and reliable results. Moreover, other research designs such as experimental technique should be utilized to test the research model.

Another area for later studies is the cultural impact surrounding these mechanisms. As societies with high power distance, individuals are more probable to take similarity to competitor’s offer as threatening to their self-worth and image, which becomes the focal reason for brand hate.

Last, studies in this area can also utilize full longitudinal designs where all variables are tapped simultaneously at each time period. Present research acknowledges further assessment on the negative side of predictors and outcomes of brand hate and similarity to the competitor’s offer.

## Conclusion

In conclusion, the present research employs a comprehensive theoretical and empirical process to examine how, why, and when brand hate can be most deleterious for brands. By utilizing the Sternberg (2003) duplex theory of hate framework, our results provide sufficient support for similarity with competitor’s offer as an important predictor of brand hate and brand equity for narcissistic individuals in the context of a developing country. The study concludes that implications that emerged from the empirical investigation of this study are fruitful for marketers as well as consumers of dairy products.

## Data Availability Statement

The original contributions presented in the study are included in the article/supplementary material, further inquiries can be directed to the corresponding author/s.

## Ethics Statement

The studies involving human participants were reviewed and approved by the FMS Research ethics board, International Islamic University, Islamabad. The patients/participants provided their written informed consent to participate in this study.

## Author Contributions

MH and FS presented the idea and designed the conceptual framework. MA and MWA performed the computations and data collection. AA, PP, and ZW reviewed and edited the manuscript. MU supervised the project. All authors contributed to the article and approved the submitted version.

## Conflict of Interest

The authors declare that the research was conducted in the absence of any commercial or financial relationships that could be construed as a potential conflict of interest.
